# Metabolome and Transcriptome Combined Reveal the Main Floral Volatile Compounds and Key Regulatory Genes of *Castanea mollissima*

**DOI:** 10.3390/plants13202865

**Published:** 2024-10-14

**Authors:** Xiaomeng Guo, Qianyu Yang, Lili Cheng, Guanglong Hu, Zhao Liu, Yanping Lan, Yunhe Cheng

**Affiliations:** 1 Institute of Forestry and Pomology, Beijing Academy of Agriculture and Forestry Sciences, Beijing 100093, China; gxm000312@126.com (X.G.); lily980101@126.com (L.C.); hglcau@gmail.com (G.H.); 2 Engineering & Technology Research Center for Chestnut of National Forestry and Grassland Administration, Beijing 100093, China; 3 Beijing Engineering Research Center for Deciduous Fruit Trees, Beijing 100093, China; zliu666666@126.com; 4 College of Forestry, Shenyang Agriculture University, Shenyang 110866, China; yangdayu0055@163.com; 5 College of Plant Science and Technology, Beijing University of Agriculture, Beijing 102206, China

**Keywords:** *Castanea mollissima*, flower, volatile organic compounds, GC–MS, transcriptome

## Abstract

Chestnut (*Castanea mollissima*) is an economically important forest tree species, and its flowers possess functions such as repelling mosquitoes, killing bacteria, and clearing heat. However, the regulatory mechanisms of floral volatile organic compounds (VOCs) in chestnut are still unclear. This study analyzed the contents of major volatile compounds and related gene expression levels in chestnut flowers during the initial flowering stage (IFS) and full-flowering stage (FFS) using metabolomics and transcription techniques. In total, 926 volatile compounds were detected, mainly terpenes, heterocyclic compounds, and esters. Acetylenone, styrene, and β-pinene had contents that exceeded 5% in FFS chestnut flowers. In total, 325 differential metabolites between the IFS and FFS were significantly (*p* < 0.05) enriched in the biosynthetic pathways of sesquiterpenes and triterpenes, as well as the ethylbenzene metabolic pathway. In total, 31 differentially expressed genes (DEGs) were related to terpenoid biosynthesis. There were only two DEGs related to the ethylbenzene metabolic pathway. In summary, we identified the volatile components of chestnut flowers and analyzed the changes in the contents of major volatile compounds in the flowers and the expression patterns of the related genes. The research results are helpful for understanding the regulation of VOCs in chestnut flowers.

## 1. Introduction

Flowers are the reproductive organs that are essential for sexual reproduction, and around 85% of flowering plants are pollinated by animals. Pollinators are attracted by not only the color and shape of flowers but also their scent. As the research into flowers deepens, the function of floral scent is gradually being better understood. The scents of flowers not only serve to attract pollinators but can also help to resist the invasion of pests and diseases, as well as facilitate communication between plants [1,2]. Humans can extract floral aromatic compounds for the production of spices and essential oils. The special components in floral odor substances can also be used in pharmaceutical production [2].

Floral scents are shaped by volatile organic compounds (VOCs). Most floral odors are sweet, typically attributed to VOCs including terpinolene, α-terpinene, and linalool [3]. A few floral scents are considered fetid due to the sulfurous and/or nitrogenous compounds among the VOCs present [4,5,6]. For example, *Castanea mollissima*, *Photinia serrulata*, *Castanopsis sclerophylla*, and *Stemona japonica* have a semen-like odor due to the presence of 1-pyrroline, 1-piperideine, 2-pyrrolidone, and phenethylamine [7,8]. More than 1700 floral VOCs have been identified so far, most of which are lipophilic liquids with low molecular weight and high vapor pressure at ambient temperatures. Floral VOCs are mainly terpenes, phenylpropanoids/benzene ring compounds, and fatty acid derivatives. Even though more and more VOCs are being identified, little is known regarding their biosynthesis pathways.

Chinese chestnut (*Castanea mollissima*) has been cultured in many countries as an important economic forest tree species because of its delicious and nutritious nuts as well as its excellent resistance to disease [9]. The *C. mollissima* flower is unisexual, and the ratio of female to male flowers is about 1:2900–3400 [10]. The large number of male flowers leads to the excessive consumption of the tree’s nutrients, limiting the improvement of the *C. mollissima* nut yield. The male flowers of *C. mollissima* have attracted more and more attention due to their antibacterial, detoxifying, dehumidifying, and lung moisturizing properties in medicine [9]. The special functions of the male flower of *C. mollissima* are mostly determined by its VOCs. Thus, studying the aroma components of chestnut flowers, and understanding the aroma components and their content changes during the flowering process of chestnut flowers, is of great significance for studying the mechanism of aroma formation and the deep development and application of chestnut flowers in food and medicine. Only about 19 VOCs have been identified in the male flowers of *C. mollissima* [7]. This cannot meet the needs of identifying the functional active ingredients, such as for the antibacterial, detoxifying, humidifying, etc., properties of chestnut flowers. Headspace Solid-Phase Microextraction–Gas Chromatography–Mass Spectrometry (HS-SPME–GC–MS) analysis can comprehensively detect volatile substances. In this study, we used HS-SPME–GC–MS technology and transcriptome sequencing to analyze the VOCs and the related biosynthesis genes of chestnut flowers at different stages. The research results will comprehensively reveal the VOC components and the synthesis pathways of the main VOCs of chestnut flowers.

## 2. Results

### 2.1. Floral Volatiles in C. mollissima

**Figure 1:**
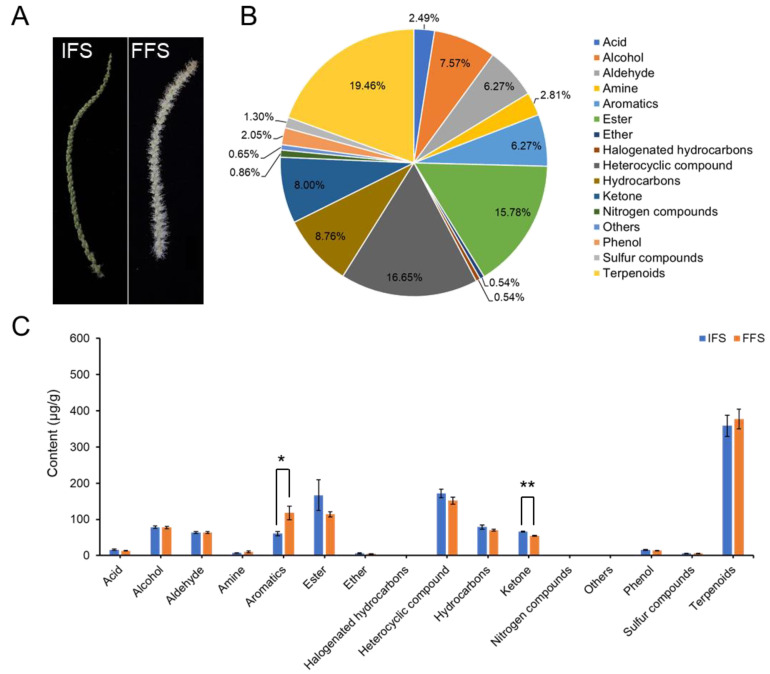
(**A**) The flowers of *C. mollissima* in the initial flowering (IFS, inflorescence 5–15% open) and full flowering (FFS, inflorescence 75–100% open) stages; (**B**) the proportion of the number of volatile substances in each category to the total number of volatile substances; and (**C**) the contents of various volatile compounds. Data represent the mean ± S.D. (*n* = 3). * *p* < 0.05, ** *p* < 0.01.

Floral volatiles of *C. mollissima* were identified at the initial flowering stage (IFS) and full flowering stage (FFS) (Figure 1A). A total of 926 volatile compounds were detected in the flowers during the initial and peak flowering periods (Table S1). These substances can be divided into 16 categories, which include 180 terpenes, 154 heterocyclic compounds, 146 esters, 81 hydrocarbons, 74 ketones, 70 alcohols, 58 aromatic hydrocarbons, 58 aldehydes, 23 acids, 26 amines, 19 phenols, 12 sulfur-containing compounds, 8 nitrogen-containing compounds, 5 halogenated hydrocarbons, 5 ethers, and 6 other substances (Table S1). The VOCs were mainly composed of terpenes, heterocyclic compounds, and esters, accounting for 51.7% of the total VOCs (Figure 1B).

Except for the significant increase in the total content of aromatic substances, from 60.53 ± 6.15 μg/g in the IFS period to 117.92 ± 18.78 μg/g in the FFS period, and the significant decrease in the total content of ketone volatile substances, from 65.9 ± 1.27 μg/g in the IFS period to 54.3 ± 1.41 μg/g in the FFS period, there were no significant differences in the total contents of other types of volatile substances between the IFS and FFS periods (Figure 1B).

### 2.2. The Changes in VOCs between the IFS and FFS in C. mollissima

**Figure 2:**
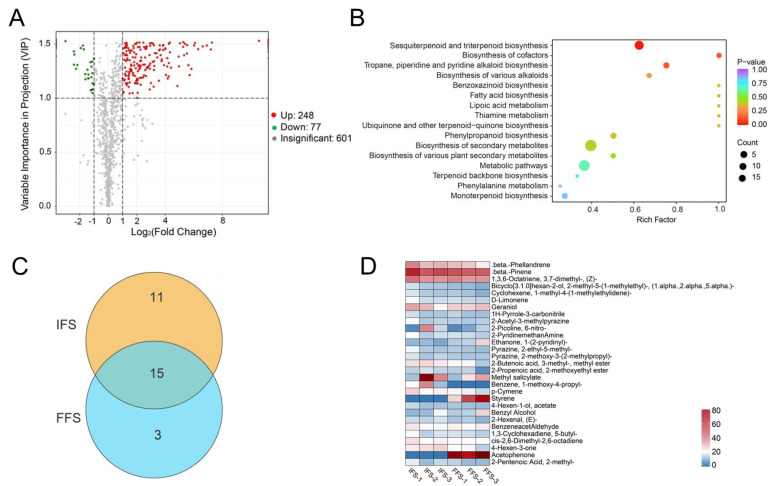
Analysis of volatile substance differences in chestnut flowers in different periods. (**A**) shows the differential volatile matter volcano map between the IFS and FFS. The red points and green points stand for volatiles which were up-regulated and significantly (*p* < 0.05) down-regulated at the FFS stage, respectively. (**B**) shows the KEGG pathway enrichment analysis of differential volatiles between the IFS and FFS. The Rich Factor is the ratio of the number of differential metabolites in the corresponding pathway to the total number of metabolites annotated for that pathway, with a higher value indicating a greater degree of enrichment. The size of the dots represents the number of significantly different metabolites enriched in the corresponding pathways. The top 16 pathways ranked by *p*-value are displayed. (**C**) Venn diagram of VOC content > 1% in Chinese chestnut flowers during the IFS and FFS. (**D**) Heatmap of major volatile substance content between the IFS and FFS.

Differential metabolites between the IFS and FFS stages were determined by VIP (VIP > 1) and absolute Log2(Fold Change) (|Log2FC| ≥ 1.0). A total of 325 significant (*p* < 0.05) differential VOCs (DEVs) were screened in the two flowering stages of chestnut (Figure 2A, Table S2). Among them, 248 substances were up-regulated and 77 substances were down-regulated during the flowering process. The up-regulated volatile substances were mainly terpenes, while the down-regulated substances included esters and terpenes (Table S2). The 325 differential metabolites were enriched in 47 pathways (Table S3), mainly including metabolic pathways (ko01100, 19 DEVs), microbial metabolism in diverse environments (ko01120, 19 DEVs), biosynthesis of secondary metabolites (ko01110, 18 DEVs), degradation of aromatic compounds (ko01220, 14 DEVs), and sesquiterpenes and triterpenoids pathways (ko00909, 10 DEVs). The sesquiterpene and triterpenoid pathways and ethylbenzene degradation pathway were significantly enriched (*p* < 0.05), which may be the main pathways for the changes between the IFS and FFS (Figure 2B).

Among all detected VOCs, 29 had a content greater than 1% (Table S4). These 29 substances accounted for over 50% of the total substance content (IFS 56.16%, FFS 54.40%). Among them, 15 substances had contents greater than 1% in both the IFS and FFS periods; 11 substances mainly existed in IFS chestnut flowers, including benzene, 1-methoxy-4-propyl, pyrazine, 2-methoxy-3-(2-methylpropyl), 2-propenoic acid, and 2-methoxyethyl ester, while acetophenone, styrene, and ethanone 1-(2-pyridinyl) mainly existed in FFS chestnut flowers (Figure 2C). Among the 29 main VOCs, only acetophenone and styrene showed significant differences between the IFS and FFS (Table S2, Figure 2D). IFS chestnut flowers contained almost no styrene and acetophenone. During the FFS, the contents of acetophenone and styrene in chestnut flowers increased sharply, to 130.31 μg/g and 67.06 μg/g, accounting for 10.25% and 5.14%, respectively, of the total VOC content (Figure 2D, Table S2).

### 2.3. The Transcriptome Analysis of C. mollissima Flowers at the IFS and FFS

**Figure 3:**
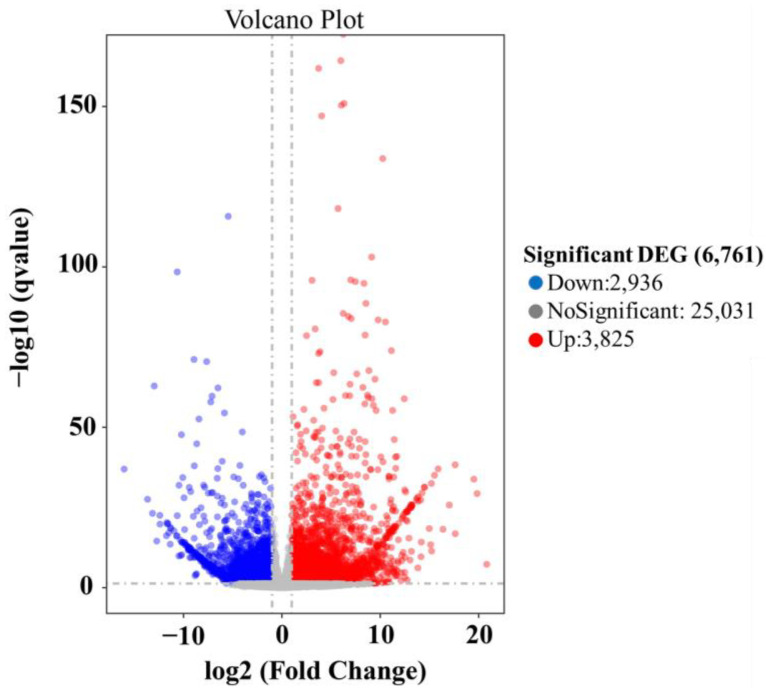
Volcano plot of differentially expressed genes between the IFS and FFS.

Through gene expression analysis, a total of 6761 differentially expressed genes were screened during the initial and peak flowering periods, which included 2936 up-regulated genes and 3825 down-regulated genes (Figure 3, Table S5). The DEGs were enriched in 151 KEGG pathways, including the biosynthesis of monoterpenoids (ko00902), diterpenoid biosynthesis (ko00904), phenylpropanoid biosynthesis (ko00940), ubiquinone and other terpenoid quinone biosynthesis (ko00130), phenylalanine metabolism (ko00360), sesquiterpenoid and triterpenoid biosynthesis (ko00909), terpenoid backbone biosynthesis (ko00900), flavonoid biosynthesis (ko00941), and tyrosine metabolism (ko00350); see Table S6.

### 2.4. Expression Analysis of Genes Related to Terpenoid Synthesis and Metabolism in C. mollissima

**Figure 4:**
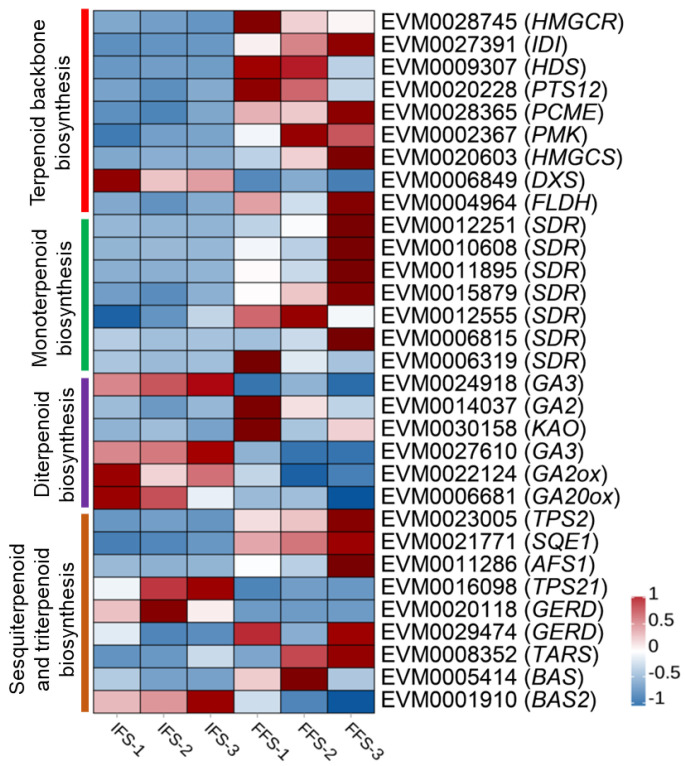
Heat map of expression of genes involved in terpenoid biosynthesis.

Terpenoid compounds mainly involve four KEGG processes, including the terpenoid backbone synthesis pathway (ko00900), monoterpenoid biosynthesis (ko00902), diterpenoid biosynthesis (ko00904), and sesquiterpenoid and triterpenoid biosynthesis (ko00909). A total of 31 differentially expressed genes related to terpenoid biosynthesis were screened (Figure 4). The terpenoid backbone synthesis pathway is the initial step for the synthesis of various terpenoid compounds. The terpenoid backbone synthesis pathway consisted of the MVA (Mevalonate) pathway and MEP (2-C-methyl-D-erythritol 4-phosphate) pathway [11,12].

In the MVA pathway, Acetyl-CoA biosynthesizes IPP (Isopentenyl-PP ) though the enzymes of AACT (Acetyl-CoA acetyltransferase 1), HMGS (Hydroxymethylglutaryl-CoA synthase), HMGR (3-hydroxy-3-methylglutaryl-coenzyme A reductase), MK (Mevalonate kinase), PMK (Phosphomevalonate kinase), and PPMD (Diphosphomevalonate decarboxylase) [11]. In this study, *HMGCS* (*EVM0020603*), *HMGCR* (*EVM0028745*), and *PMK* (*EVM0002367*) were up-regulated during the FFS period. In the MEP pathway, Pyruvate and GA-3-P biosynthesize Dimethylallyl-PP (DPP) though the enzymes of DXS (1-deoxy-D-xylulose-5-phosphate synthase), DXR (1-deoxy-D-xylulose 5-phosphate reductoisomerase), MCT (2-C-Methyl- D-erythritol 4-phosphate), CMK (4-(Cytidine 5’-diphospho)-2-C-methyl-D-erythritol), MDS (2-Phospho-4-(cytidine 5’-diphospho)-2-C-methyl-D-erythritol), HDS (2-C-Methyl- D-erythritol 2,4-cyclodiphosphate), and HDR (4-Hydroxy-3-methylbut-2-enyl-diphosphate). Only *DXS* (*EVM0009849*) and *HDS* (*EVM0009307*) were differentially expressed between the IFS and FFS stages, and *DXS* was down-regulated at the FFS stage, while *HDS* was up-regulated at the FFS stage. IPP and DPP convert under the action of IDI (isopentenyl-diphosphate Delta-isomerase). In this study, *IDI* (*EVM0027391*) expression was up-regulated during the FFS stage.

Subsequently, IPP was transformed into FPP (Farnesyl-PP). FPP synthesizes sesquiterpenoids and triterpenoids or converts to trans-farnesol under the action of PCME (prenylcysteine alpha-carboxyl methylesterase). Trans-farnesol can also be transformed into FPP under the action of FLDH (NAD(P)-binding Rossmann-fold superfamily protein). The expression of *PCME* (*EVM0028365*) and *FLDH* (*EVM0004964*) were also up-regulated at the FFS stage. In the MEP pathway, DPP firstly transformed into GPP (Geranyl diphosphate), and then synthesizes diterpenoid [11]. The first key enzyme gene *Probable terpene synthase* 12 *PTS12* (*EVM0020228*) involved in the GPP synthesis of monoterpenoids was up-regulated at the FFS stage. In the monoterpenoids synthesis pathway, there were seven (+)-Neomenthol biosynthesis gene *(+)-neomenthol dehydrogenase SDRs*, all of which are up-regulated during the FFS stage. There are six differentially expressed genes in the diterpenoid biosynthesis pathway, among which the key genes *Ent-kaur-16-ene synthase GA2* (*EVM0014037*) and *Ent-kaurenoic acid oxidase KAO* (*EVM0030158*) for GA12 synthesis are up-regulated during the FFS stage, while the expression levels of *Ent-kaurene oxidase GA3* (*EVM0024918*, *EVM0027610*), *Gibberellin 2-beta-dioxygenase GA2ox* (*EVM0022124*), and *Gibberellin 20 oxidase GA20ox* (*EVM0006681*) are down-regulated. Six differentially expressed genes were upregulated in the sesquiterpenoid and triterpenoid biosynthesis pathway, including *Probable alpha,alpha-trehalose-phosphate synthase [UDP-forming] 2 TPS2* (*EVM0023005*), *Squalene epoxidase 1 SQE1* (*EVM0021771*), *(E,E)-alpha-farnesene synthase AFS1* (*EVM0011286*), (-)-*germacrene D synthase GERD2* (*EVM0029474*), *Taraxerol synthase TARS* (*EVM0008352*), and *Beta-amyrin synthase BAS* (*EVM0005414*). *TPS2* and *GERD2* (*EVM0029474*) are responsible for synthesizing germacrene-type sesquiterpenoids, while *AFS1* is a key gene for acyclic sesquiterpenoids. *SQE1*, *TARS*, and *BAS* are responsible for the biosynthesis of triterpenoid. Three genes, *TPS21* (*EVM0016098*), *GERD1* (*EVM0020118*), and *BAS2* (*EVM0001910*), are downregulated in expression. Among them, *TPS21* and *GERD1* (*EVM0020118*) are genes involved in the synthesis of germacrene-type sesquiterpenoids, while *BAS2* (*EVM0001910*) is responsible for the biosynthesis of triterpenoid.

### 2.5. Expression Analysis of Acetophenone- and Styrene-Related Genes in C. mollissima

**Figure 5:**
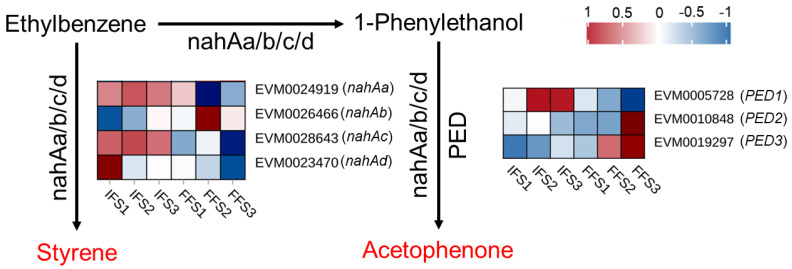
The expression analysis of acetophenone and styrene biosynthesis genes. Styrene and acetophenone in red mean the content of the two VOCs increased at FFS stage.

During the FFS period, acetophenone and styrene were the most abundant VOCs in chestnut flowers, both of which are involved in the ethylbenzene degradation pathway (Ko00642) [13]. Seven genes including *3-oxoacyl-[acyl-carrier-protein] reductase PED* (*EVM0019297*, *EVM0005728*, *EVM0010848*), *nahAa* (*EVM0023470*, *EVM0026466*, *EVM0028643*), and *nahAb* (*EVM0024919*) are related to the synthesis of acetophenone and styrene in the ethylbenzene degradation pathway (Figure 5). The synthesis gene *PED* (*EVM0019297*) of acetophenone was significantly increased in the FFS, while *nahAa* (*EVM0028643*) was significantly decreased (Figure 5).

## 3. Discussion

The types and contents of volatiles in flowers determine the aromas and functions of flowers. To date, VOCs in the Chinese rose and lily plants have been determined through headspace microextraction and gas chromatography–mass spectrometry [14,15,16,17]. A total of 47 VOCs were detected in both the rose [14] and lily [15], 43 VOCs were detected in *Nymphaea hybrid* [16], and 53 VOCs were detected in *Panax notoginseng* [17]. In this study, a total of 926 volatile compounds were detected in *C. mollissima*, which is much higher than that in other plants. This may have been determined by the sampling and testing methods. For the volatile compounds of lily and Chinese rose flowers, fresh samples were used, and the samples were ground to determine their volatile components directly [14,15,16,17]. Only 21 volatile compounds could be identified in chestnut flowers without grinding the samples [7]. The Japanese chestnut inflorescence was determined after the extraction of essential oils, and 72 volatile substances with a content greater than 0.01% were identified [18]. In this study, chestnut flowers were frozen in liquid nitrogen and ground into powder, then put into a headspace bottle, dissolved in saturated NaCl, and shaken at 60 °C for 5 min. Then, the volatile components were detected. This method allows for the identification of more volatile substances, which is beneficial for the study of the anabolic processes of volatile substances [19,20,21].

In this study, the VOCs of chestnut flowers were mainly terpenes, heterocycles, esters, hydrocarbons, ketones, alcohols, aromatic hydrocarbons, and other substances, which are similar to those of most other plants [15,22,23]. During the FFS period, the relative contents of acetophenone and styrene in the metabolic pathways of β-pinene and ethylbenzene, which are the main components of the Chinese chestnut’s floral fragrance, were all greater than 5%. This result is similar to the VOCs in chestnut and Japanese chestnut (*Castanea crenata*) flowers reported in other studies [7,18]. In *C. crenata*, acetophenone and styrene were some of the main VOCs of the flowers. Acetophenone and its derivatives are the characteristic components of the *C. crenata* floral fragrance [18]. The volatile substances in the flowers of plants such as honeysuckle, chrysanthemum, peony, and dendrobium have the effects of killing bacteria, inhibiting bacteria, and deterring pests. Chestnut flowers have both excellent antioxidant and antimicrobial properties and have been used to make deworming, antibacterial, and medicinal products [9,24,25,26]. Among the major volatile components of chestnut flowers identified in this study, β-pinene has been shown to have mosquito repellent, expectorant, and antitussive effects [27,28,29]. Acetophenone can be used to synthesize some analgesic, antiviral, anticancer, and antidepressant drugs [30]. Styrene is genotoxic, and styrene oxide—the major in vivo metabolite of styrene—is listed as a possible carcinogen [31,32]. However, the hazards of styrene to insects are not known. Our results imply that β-pinene and acetophenone may be the main components of chestnut flower responsible for its anthelmintic, antibacterial, and medical properties.

The species and contents of volatiles in flowers vary with the plant species and flower development stages [15,33]. There are usually many kinds of VOCs in flowers in full bloom, and large amounts of volatiles are released [34]. There were 325 differential metabolites between chestnut flowers in the FFS and IFS. These differential VOCs were significantly enriched in sesquiterpenoid and triterpenoid biosynthesis and ethylbenzene metabolism pathways. Terpenoids, including monoterpenes (C10), sesquiterpenes (C15), and polyterpenes, are the most diverse natural products in nature and are also the most intensively studied compounds around the world. Plants first synthesize the terpenoid precursors dimethylallyl diphosphate (FPP) and isoprene pyrophosphate (GPP) through two pathways: mevalonic acid (MVA) and methylerythritol (MEP) [11,12]. FPP is further used to synthesize sesquiterpenoid and triterpenoids, while GPP further serves to synthesize monomers and diterpenoids. The differential metabolites in the IFS and FFS in this study were mainly sesquiterpenoids and monoterpenoids, and all terpenoids were up-regulated except for (E, E)-Farnesol. HMGCS, HMGR, MK, and PPMD are the key enzymes of the MVA pathway for the synthesis of terpenoid skeletons [11], while DXS, DXR, MCT, CMK, MDS, HDS, and HDR are the key enzymes for the synthesis of terpenoid skeletons in the MEP pathway [11]. The expression levels of these key enzyme genes gradually increase during flowering and decrease after full flowering [35,36,37]. In this study, the expression levels of differentially expressed genes in the terpenoid skeleton synthesis pathway were up-regulated in the FFS except for DXS. These results indicate that the synthesis of terpenoids gradually increased during the flowering process of chestnut, further confirming the conclusion that terpenoids are the main components of chestnut volatiles.

**Figure 6:**
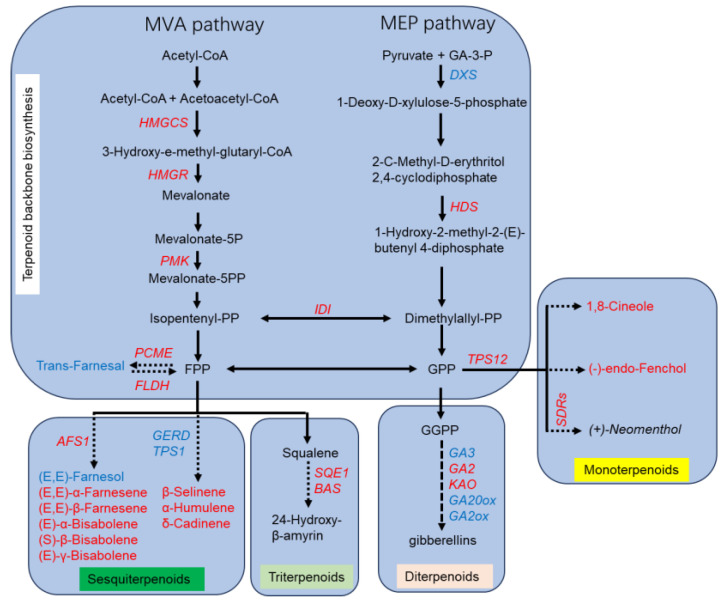
Schematic diagram of terpenoid synthesis pathway from the first flowering stage to the full flowering stage of chestnut. Red indicates the up-regulated genes or VOCs. Blue indicates the down-regulated genes or VOCs.

*DXS* is the first transketolase in the MEP pathway and also the highest rate-limiting enzyme [38]. The overexpression or inhibition of *DXS* can cause changes in the contents of downstream metabolites. Silencing the *SIDXS2* gene in tomato caused a decrease in the β-phellandrene content [39]. However, the activity of *DXS* is also subject to feedback regulation by 3-isopentenyl pyrophosphate (IPP) and dimethylallyl pyrophosphate (DMAPP). IPP and DMAPP will competitively bind to the active center of the DXS enzyme and disengage TPP, thereby inhibiting the activity of the DXS enzyme [40]. In this study, there was no significant difference in each product of the terpenoid backbone synthesis pathway from the IFS to FFS; however, the contents of sesquiterpenoids, monomers, and synthetic genes were up-regulated in the FFS (Figure 6). This suggests that substances in the DMEP pathway do not decrease the activity of the DXS enzyme during chestnut blooming, which may be the reason for the decreased expression of *DXS* in the FFS.

Acetophenone has a bactericidal function and has been applied in the disease control of plant cultivation and harvested agricultural products. After fumigation with 100 μL/L acetophenone for 12 h, it showed good antifungal activity against the anthracnose pathogen in postharvest chili fruit [41]. Acetophenone showed antifungal activity to *Penicillium italicum* [42]. The currently used acetophenone mainly comes from artificial synthesis. In fact, acetophenone could be biosynthesized through styrene metabolic pathway in bacteria such as the *Azoarcus*-like strain EbN1 and *Streptomyces globisporus* JK-1 [13,42]. However, the endogenous biosynthesis pathway of acetophenone in plants is still unclear. Acetophenone was not detected in the IFS period of chestnut, and the styrene content was less than 0.01%. However, during the FSS period, the styrene and acetophenone contents increased sharply, to more than 10% and 5%, respectively. This result indicates that acetophenone can also be synthesized in plants. During the FFS stage, acetophenone is the most abundant VOCs, suggesting that acetophenone may be one of the main active ingredients in the sterilization and disinfection function of chestnut inflorescences.

Ethylbenzene is catalyzed by *nahAa/b/c/d* to form styrene or 1-Phenylethanol. 1-Phenylethanol forms acetophenone under the combined action of *nahAa/b/c/d* and *PED* [13,43]. Among the genes related to bacterial acetophenone and styrene synthesis in this study, the *PED* (EVM0019297) and *nahAa* (*EVM0028643*) genes were up-regulated and down-regulated, respectively, during the FFS. *PED* is a specific gene for acetophenone synthesis, and *nahAa* is a common gene for acetophenone and styrene synthesis. This suggests that the *nahAa* homolog in chestnut may function to promote styrene synthesis and inhibit acetophenone synthesis. However, the functions of chestnut *PED* and *nahAa* need to be further verified. Meanwhile, the synthetic pathways of acetophenone and styrene in chestnut require further elucidation.

## 4. Materials and Methods

### 4.1. Materials

Three *Castanea mollissima* individuals with a diameter at breast height of about 15 cm and a height of 3.6 m from the chestnut high-efficiency cultivation demonstration base of the Forestry and Fruit Research Institute of Beijing Academy of Agricultural and Forestry Sciences were selected as the three biological replicates for sampling. On 18 May 2022 (initial flowering stage: inflorescence opening 5% to 15%) and 7 June 2022 (full-flowering stage: inflorescence opening 75% to 100%) (Figure 1), 10 male inflorescences were randomly selected from each tree for sampling. The collected samples were quickly placed in a 50 mL centrifuge tube (sealed), frozen in liquid nitrogen, and stored in a −80 °C freezer for subsequent experiments.

### 4.2. Headspace Solid-Phase Microextraction–Gas Chromatography–Mass Spectrometry (HS-SPME–GC–MS)

Chestnut male flowers were ground into powder by adding them to liquid nitrogen in a mortar, vortexing, and mixing evenly. Then, 500 mg of the powder was transferred immediately to a 20 mL head-space vial (Agilent, Palo Alto, CA, USA) containing 2 mL of saturated NaCl solution to inhibit any enzyme reaction. The vials were sealed using crimp-top caps with tetrafluoroethylene (TFE)-silicone headspace septa (Agilent). At the time of SPME analysis, each vial was placed at 60 °C for 5 min, and then a 120 µm DVB/CWR/PDMS fiber (Agilent) was exposed to the headspace of the sample for 15 min at 60 °C. A total of 20 μL (10 μg/mL) of internal standard (3-Hexanone-2,2,4,4-d4) solution was added separately to the headspace vial, which was then sealed. Fully automated headspace-solid phase microextraction (HS-SPME) was simultaneously performed for sample extraction for GC–MS analysis.

The identification and quantification of VOCs were carried out using an Agilent Model 8890 GC and a 7000D mass spectrometer (Agilent) equipped with a 30 m × 0.25 mm × 0.25 μm DB-5MS (5% phenyl-polymethylsiloxane) capillary column. The GC conditions were consistent with the work of Yuan [19]. Helium was used as the carrier gas, at a linear velocity of 1.2 mL/min. The injector temperature was kept at 250 °C, and the detector temperature was kept at 280 °C. The oven temperature was programmed from 40 °C (3.5 min), increasing at 10 °C/min to 100 °C, at 7 °C/min to 180 °C, at 25 °C/min to 280 °C, and held for 5 min. Mass spectra were recorded in electron impact (EI) ionization mode at 70 eV. The quadrupole mass detector, ion source, and transfer line temperatures were set to 150, 230, and 280 °C, respectively. The MS was set in ion monitoring (SIM) mode for the identification and quantification of analytes. Volatile components were identified by comparison of the retention index (RI, determined by n-alkanes C7-C40) and the National Institute of Standards and Technology (NIST) mass spectral database in the NIST library (https://www.nist.gov/, accessed on 15 July 2024).

The formula for calculating the relative content (µg/g) of volatile components is as follows [44]:

$$\mathrm{Content}\;\left(µg/g\right)=\frac{\mathrm{target}\;\mathrm{volatile}\;\mathrm{component}\;\mathrm{peak}\;\mathrm{area}\times \mathrm{internal}\;\mathrm{standard}\;\mathrm{mass}\;\left(µg\right)}{\mathrm{internal}\;\mathrm{standard}\;\mathrm{peak}\;\mathrm{area}\;\times \;\mathrm{sample}\;\mathrm{mass}\;\left(g\right)\;}$$

### 4.3. Analysis of Volatile Compounds (VOCs)

Differential metabolites between the IFS and FFS stages were determined according to the variable importance in projection (VIP > 1), the absolute Log2FC (|Log2FC| ≥ 1.0) values, and *p* < 0.05 in the independent samples t-test [44]. VIP values were extracted from the orthogonal partial least squares discriminant analysis (OPLS-DA) result, which also contains score plots and permutation plots, generated using the R package (3.0) MetaboAnalystR (McGill University, Montreal, Quebec, Canada).

The identified metabolites were annotated using the KEGG Compound database (http://www.kegg.jp/kegg/compound/, accessed on 10 July 2024), and annotated metabolites were then mapped to the KEGG Pathway database (http://www.kegg.jp/kegg/pathway.html, accessed on 10 July 2024). Pathways with significantly differentially regulated metabolites were then fed into MSEA (metabolite sets enrichment analysis), and their significance was determined according to the hypergeometric test’s *p*-values [44].

### 4.4. Transcriptome Sequencing and Analysis

Total RNA was extracted from chestnut flowers using a TaKaRa MiniBEST Universal RNA Extraction Kit (9769) (TaKaRa, Ōsaka, Japan). Then, 1 μg of total RNA was used for library preparation. Poly(A) mRNA isolation was performed using Oligo(dT) beads, and mRNA fragmentation was performed using divalent cations. cDNA were synthesized using NEBNext UltraTM RNA Library Prep Kit for Illumina (NEB, Ipswich, MA, USA) following manufacturer’s recommendations. The purified double-stranded cDNA was then treated to repair both ends and add a dA tail in a single reaction, followed by a T-A ligation to add adaptors to both ends. Size selection of adaptor-ligated DNA was then performed using DNA Clean Beads. Each sample was then amplified via polymerase chain reaction (PCR) using P5 and P7 primers, and the PCR products were validated. Libraries with different indices were then multiplexed and loaded on an Illumina HiSeq 3000 (Illumina, San Diego, CA, USA) instrument for sequencing using a 2 × 150 paired-end (PE) configuration according to the manufacturer’s instructions.

We performed quality control using fastp to ensure meaningful downstream analysis: (1) Discard paired reads with adapter contamination (>10 aligned nucleotides, ≤10% mismatches). (2) Discard reads with >10% uncertain bases. (3) Discard reads with >50% low-quality bases (Phred < 5). (4) Discard reads <20 nucleotides after trimming [45]. Downstream analyses were conducted on the resulting high-quality data. QC statistics (total reads, raw data, raw depth, error rate, and Q30 percentage) were calculated. Reference genome sequences and gene model annotation files of *C. mollissima* were downloaded from http://www.chestnutgenome.cn/#/download (accessed on 7 July 2024). Hisat2 (v2.2.1) was used to index reference genome sequence. Clean data were aligned to the reference genome via the Hisat2 (v2.2.1) software. HTSeq (v0.6.1) was used to estimate the raw counts of genes and isoforms and then calculate the FPKM of each gene based on the gene length. DESeq2 (V1.26.0) was used for differential gene analysis, with the following criteria: padj (FDR) < 0.05, | fold change | > 1.5, and *p*-value < 0.005 for screening.

Gene Ontology (GO) and KEGG pathways enrichment analysis of differential expressed genes was implemented by the clusterProfiler R package (3.8.1). Terms with corrected *p*-values less than 0.05 were considered significantly enriched by differentially expressed genes. In this part, all the annotated genes of *C. mollissima* were used for the background of the enrichment analysis.

## 5. Conclusions

The VOCs of Chinese chestnut flowers were mainly terpenoids, heterocycles, esters, hydrocarbons, and ketones. Acetophenone, β-pinene, and styrene were the main components of the chestnut flower fragrance. Most of genes related to the terpenoids biosynthesis pathway and the biosynthesis pathway of acetophenone and styrene were up-regulated during the flowering process.

## Data Availability

The original contributions presented in the study are included in the article/supplementary material, further inquiries can be directed to the corresponding author/s.

## Supplementary Materials

The following supporting information can be downloaded at: https://www.mdpi.com/article/10.3390/plants13202865/s1. Table S1: The identified VOCs in *Castanea mollissima* flower; Table S2: Differential VOCs between IFS and FFS; Table S3: KEGG enrichment of differential VOCs between IFS and FFS; Table S4: The main VOCs that content more than 1%; Table S5: Differential expression genes between IFS and FFS; Table S6: Enriched KEGG pathways of differential expression genes between IFS and FFS.
